# Optical mode-controlled topological edge state in waveguide lattice

**DOI:** 10.1515/nanoph-2023-0680

**Published:** 2024-01-23

**Authors:** Changyu Zhou, Zhenwei Xie, Ting Lei, Yao Zhang, Qinmiao Chen, Xiaocong Yuan

**Affiliations:** Nanophotonics Research Center, Institute of Microscale Optoelectronics & State Key Laboratory of Radio Frequency Heterogeneous Integration, Shenzhen University, Shenzhen 518060, China; State Key Laboratory on Tunable Laser Technology, Ministry of Industry and Information Technology Key Lab of Micro-Nano Optoelectronic Information System, Harbin Institute of Technology (Shenzhen), Shenzhen, China; Research Institute of Intelligent Sensing, Research Center for Humanoid Sensing, Zhejiang Lab, Hangzhou 311100, China

**Keywords:** SSH model, waveguide lattice, topological edge state, topological mode splitter

## Abstract

Topological edge state (TES) has emerged as a significant research focus in photonics due to its unique property of unidirectional transmission. This feature provides immunity to certain structural disorders or perturbations, greatly improving the robustness of photonic systems and enabling various applications such as optical isolation and topological lasers. Nevertheless, most of current researches focus on the fixed generated TES with no means to control, leaving untapped potential for manipulating the TES through specific methods. In this work, we propose a topological Su–Schriffer–Heeger (SSH) waveguides-lattice scheme that enables the controllable TES without changing the topological phase of the system. Light is selectively localized at the edges of the SSH waveguide lattice, which is determined by the special waveguide modes. Eventually, achieving an effective mode splitter. To validate our proposal, we further demonstrate such mode-controlled TES with a fabricated on-chip device in experiment. The experimentally tested results confirm a successful separation of the waveguide modes with the mode extinction ratio of approximately 10 dB in each channel near the wavelength of 1550 nm. This scheme presents a promising approach for manipulating the TES in photonic systems, thereby facilitating the design of optical controllable topological photonic devices.

## 1 Introduction

Recently developed topological photonics has opened up a new avenue for simulating various topological effects observed in condensed matter physics [1]. One of the most notable features is the emergent photonic topological edge state (TES) in a topological system [2], which has garnered significant research attention in recent years after the seminal works by Haldane and Raghu [3], [4]. These works theoretically predicted the existence of robust TES using the two-dimensional magneto-optical periodic elements with broken time-reversal symmetry. The emergent TES possesses the peculiarity of resisting disorders or perturbations of the system, which deeply roots in the nontrivial topological invariant (Chern number) in the photonic bands. Subsequently, this phenomenon was experimentally demonstrated using magneto-optical photonic crystals in the microwave range [5]. However, a major challenge is the lack of large magneto-optical responsive materials in optical range. Soon after, Photonic TES was also achieved and verified extensively in various optical platform, such as metamaterials [6], [7], [8], waveguide arrays [9], [10], [11], [12], [13], and ring waveguides lattices [14], [15], [16], [17], [18], [19], beyond photonic crystal.

Remarkably, photonic TES can also arise in one-dimensional topological systems, such as Su–Schriffer–Heeger (SSH) model [20], [21], [22], [23] and topological pump model [9], [24], [25], [26], [27]. These models have been implemented in photonic waveguides platform with the advantage of visualizing the time-like evolution of states along the waveguide, providing a feasible scheme to achieve a series of applications in photonics, including topological lasers [28], [29], [30], optical isolator [31], [32], waveguide mode convertor [24], topological beam splitter [33], and directional coupler [20], [34], demonstrating great potential for various applications in photonic system.

Nevertheless, most of current researches focus on a fixedly generated TES. Several topological schemes have been proposed to alter the topological phase with the utilization of such as light frequency [18], [35] and polarization [35]. It is noteworthy that these manipulations induce the trivial bulk states, which is unadaptable for applications. In addition, frequency manipulation can be easily achieved owing to the frequency dispersion in the photonic system, the utilization of other manipulation dimensions remains to be exploited, and can open up the possibility of extending the opportunity in controllable topological photonic device design. In this work, we propose a topological SSH waveguide-lattice scheme to achieve the mode-selected TES, without changing the topological phase of the system. Light is mainly localized at one of the edges in the SSH waveguide lattice for the input basic TE_0_ mode, while it is directionally coupled to another edge for the input TM_0_ mode, achieving equivalently a topological mode splitter [36]. We have also experimentally demonstrated the mode-controlled TES with a fabricated device in the telecom-wavelength range. The experimental results exhibit an evident transmission signature along the edge with approximately 10-dB mode extinction ratio in each channel near the wavelength of 1550 nm. This work may pave the way for controllable topological photonic devices design.

## 2 Device design

**Figure 1:**
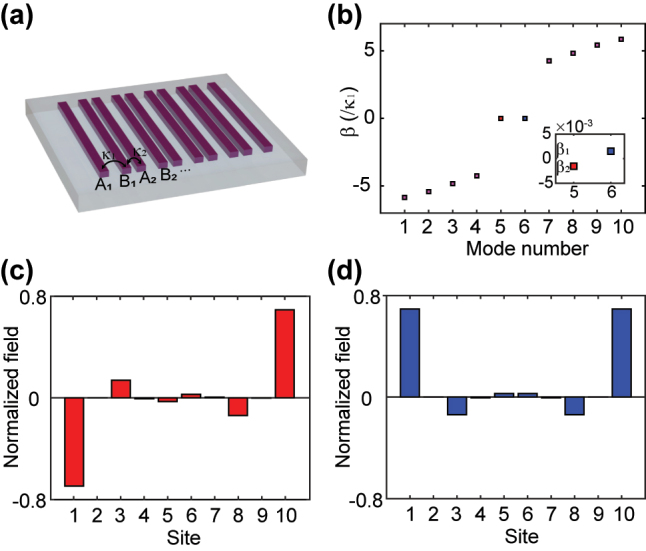
Analyses for the SSH model. (a) Schematic of the SSH waveguide lattice. (b) Band structure of the ten-sites SSH model. The inset shows the amplified near-zero eigenvalues *β*_1_ and *β*_2_ of the TESs, and *κ*_2_/*κ*_1_ = 5 is used. (c–d) The calculated TESs for the near-zero eigenvalues of (c) *β*_2_ and (d) *β*_1_ in (b).

The fundamental design in this work is based on the SSH model, in which the two inequivalent lattice sites are arrayed periodically along one direction. The SSH model can be described using the form of tight-binding Hamiltonian

(1) $$H=\underset{j}{\sum }\left(\kappa_{1}a_{j}^{†}b_{j}+\kappa_{2}b_{j}^{†}a_{j+1}\right)+h.c.,$$

where $a_{j}^{†}\left(a_{j}\right)$ and $b_{j}^{†}\left(b_{j}\right)$ represent the creator (annihilator) on *j*th lattice sites *A*_*j*_ and *B*_*j*_, respectively. *κ*_1_ and *κ*_2_ denote the hopping strengths in neighbor sites. In photonic systems, the SSH model has been successfully implemented in waveguide arrays (Figure 1(a)) [20], where the evanescent coupling between waveguides is introduced to simulate the hopping between the lattice sites.

The topological property of the SSH model, which is described by the winding number *W* or Zak phase [37], is dependent on the coupling (hopping) strengths *κ*_1_ and *κ*_2_ in Eq. (1). The analysis for the Hamiltonian Eq. (1) in momentum space indicates the calculated two bands are separated by the magnitude of 2*δ* = 2|*κ*_1_ − *κ*_2_|. The system is topologically trivial (*W* = 0) for *κ*_1_ > *κ*_2_, while is topologically nontrivial (*W* = 1) for *κ*_1_ < *κ*_2_ [20]. For a finite even-sites SSH model, two special modes emerge with the eigenvalues near zero in the topologically nontrivial phase for *W* = 1 (Figure 1(b)). These two modes are mostly localized at two edges of the lattice (Figure 1(c) and (d)), corresponding to a pair of TESs. Remarkably, the eigenvalues *β*_1_ and *β*_2_ of these two TESs (i.e., propagation constants of the TESs) slightly deviate the exact zero, enabling the coupling between these two TESs.

**Figure 2:**
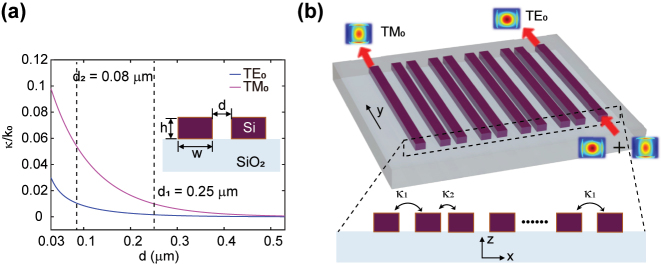
Schematic for the mode controlled edge states in the SSH waveguide lattice. (a) Simulated coupling-coefficients curves *κ*/*k*_0_ for the TE_0_/TM_0_ mode with varying the gap distance *d* between the two waveguides. The schematic is shown in the inset, detailed simulation parameters are *w* = 500 nm, *h* = 350 nm, and the simulation wavelength is 1550 nm. (b) Schematic illustration of the mode-controlled TESs.

The basic mechanism to achieve the controllable TES in this work is by exploiting the difference of coupling (DOC) *β*_*d*_ = *β*_1_−*β*_2_ between two near-zero edge modes in a finite even-sites SSH model. According to the coupled-mode theory [38], TES located at one of the edges can be directionally coupled to another edge in the SSH waveguide lattice, with the coupling length *L*_*C*_ depending on the DOC *β*_*d*_ (*L*_*C*_ = *π*/*β*_*d*_). This implies that the DOC decides the duration of TES at the edge sites in a distinct manner: a lager DOC value denotes a shorter duration of the TES at one of the edge sites. In addition, the large coupling-coefficients differences for different propagation modes in a single waveguide naturally provide a means of manipulating the TES. In Figure 2(a), the simulated coupling-coefficients curves for the TE_0_ and TM_0_ mode in a strip silicon waveguide are displayed. The chosen parameters of the coupling coefficients can exhibit significant differences for the TE_0_ and TM_0_ modes, resulting in distinguishing TESs for each mode. This ultimately achieves the mode-controlled TES in the SSH waveguide lattice. In our design, the excited TE_0_ mode is mainly localized at the input edge waveguide, whereas the TM_0_ mode is directionally coupled to another edge (Figure 2(b)), achieving equivalently a topological mode splitter.

## 3 Simulation results

**Figure 3:**
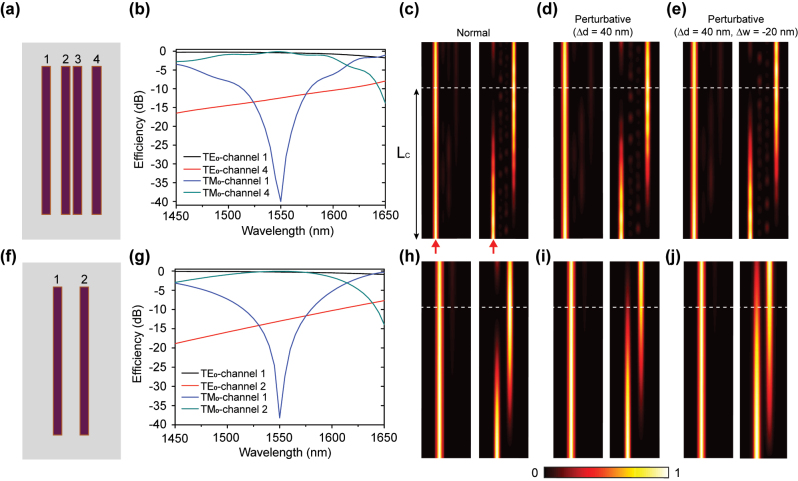
Simulation results of the propagated field distributions in the SSH waveguide lattice and their conventional counterparts. (a) Schematic of the designed four-sites SSH waveguide lattice. The corresponding waveguides (channels) are marked by the number 1–4, respectively. (b) Simulated efficiencies curves in the channels 1 and 4 under input TE_0_/TM_0_ modes. (c–e) Simulated intensity distributions at the wavelength of 1550 nm for the configurations (c) normal, (d) with perturbation Δ*d* = 40 nm and (e) with perturbation Δ*d* = 40 nm, Δ*w* = −20 nm. The red arrow and the value *L*_*C*_ in (c) indicate the input-source position and the designed coupled length for TM mode, respectively. (f) Schematic of the conventional double-waveguides structure, marked by channels 1 and 2. (g) Simulated efficiencies curves for the conventional structure in the channels 1 and 2 with input TE_0_/TM_0_ modes. (h–j) Simulated intensity distributions for the conventional configurations (h) normal, (i) with perturbation Δ*d* = 40 nm and (j) with perturbation Δ*d* = 40 nm, Δ*w* = −20 nm.

As an example, we have designed a four-sites SSH lattice with four silicon waveguides arrayed on a silica substrate (Figure 3(a)). The designed width of the silicon waveguides is 500 nm and the height is fixed at 350 nm, to allow only the TE_0_ and TM_0_ mode. To obtain a large coupling-coefficient differences for different waveguide modes, the chosen gap distances between the waveguides in the SSH lattice are *d*_1_ = 250 nm and *d*_2_ = 80 nm (Figure 2(a)). These parameters correspond to the coupling coefficients of *κ*_1_/*k*_0_ ≈ 0.0016 and *κ*_2_/*k*_0_ ≈ 0.0112 for the TE_0_ mode (*k*_0_ = 2*π*/*λ*_0_ is the wave vector in free space and *λ*_0_ = 1550 nm), and *κ*_1_/*k*_0_ ≈ 0.0099 and *κ*_2_/*k*_0_ ≈ 0.0568 for the TM_0_ mode. These values result in the coupling lengths of *L*_TE_ ≈ 1730 µm for TE_0_ mode and *L*_TM_ ≈ 230 µm for TM_0_ mode. In our design, the waveguide length is set to *L*_*C*_ = *L*_TM_ = 230 µm. Because the coupling length *L*_TE_ is much larger than *L*_TM_, the TE_0_-mode TES still resides in the input end with the efficiency ≈91 %, while the TM_0_-mode TES is directionally coupled to the other edge waveguide with an efficiency of approximately 97 %, as indicated by the simulation results in Figure 3(b). This demonstrates an effective mode splitter, as supported by the simulated field distributions shown in Figure 3(c). In contrast, these results are similar to the results in the conventional double-waveguides configuration [39], as shown in Figure 3(f)–(h), where the corresponding efficiencies for the TE_0_ and TM_0_ edge channels are 94 % and 98 %, respectively.

To analyze the structure perturbations to the SSH lattice, $\Delta d=\sum \Delta d_{i}=\sum {d^{\prime }}_{i}-d_{i}$ and Δ*w* = = *w*′ − *w* are defined to represent the total gap deviation and the waveguide-width deviation, respectively. Here $d_{i}^{\prime }\left(w^{\prime }\right)$ and *d*_*i*_(*w*) are corresponding actual and preset gap distance (waveguide width). Figure 3(d) shows the simulation results of the SSH waveguide lattice with the gap perturbation of Δ*d* = 40 nm, the function for the effective mode splitter remains unchanged, with over 90 % efficiency for both edge channels. While for the conventional configuration with same gap perturbation (Δ*d* = 40 nm) in Figure 3(i), the input TE_0_ mode still preserves the transmission in the original waveguide with a high efficiency of 96 %, whereas the directionally coupled efficiency in the TM_0_ edge channel is only 74 %, slightly lower than the SSH configuration in Figure 3(d). In addition, the mode controlled TES can also be robust to resist the waveguide-width-deviation perturbation. For the deviation of Δ*d* = 40 nm and Δ*w* = −20 nm, the efficiencies for both edge channels are approximately 90 %, as shown in Figure 3(e). In contrast, the conventional configuration with same parameter (Δ*d* = 40 nm, Δ*w* = −20 nm) in Figure 3(j), shows an efficiency of 68 % for the TM_0_ edge channel, which is also lower than that in Figure 3(e). The analyses above demonstrate the strong performance of mode-controlled TESs. In contrast to the conventional double-waveguides configuration, our calculations show that the coupled length *L*_*C*_ for directional coupling in the topological SSH waveguide lattice is less affected by variations in gap and width. This results in a system with improved robustness.

## 4 Experimental results

**Figure 4:**
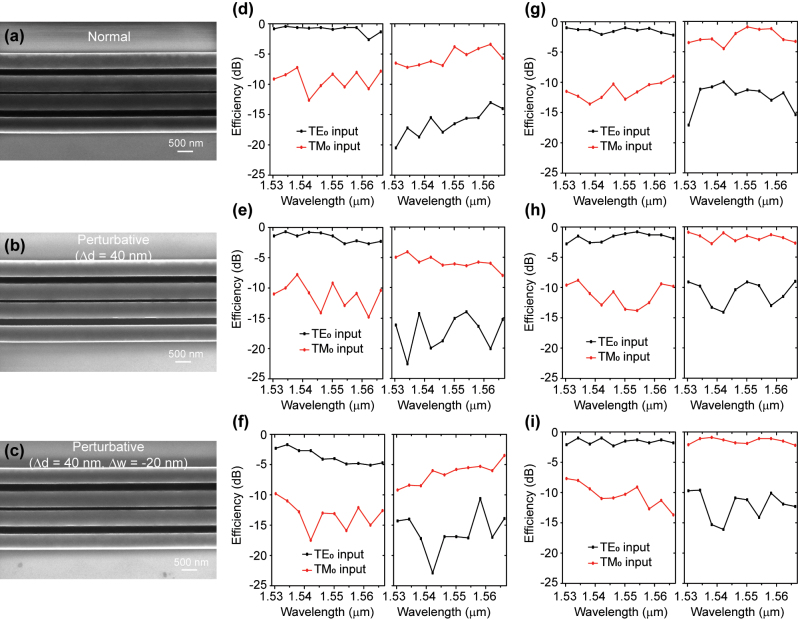
Experimentally tested efficiency curves of the two topological edge states in the SSH waveguide lattice. (a–c) SEM images of the three type of designs, (a) normal, (b) gap perturbation Δ*d* = 40 nm and (c) perturbation Δ*d* = 40 nm, Δ*w* = −20 nm. (d–f) Responsive efficiency curves detected in the TE_0_ edge channel (left subfigure) and the TM_0_ edge channel (right subfigure) for the configurations (d) normal, (e) gap perturbation Δ*d* = 40 nm and (f) perturbation Δ*d* = 40 nm, Δ*w* = −20 nm. (g–i) Responsive efficiency curves for the conventional double-waveguides configurations (g) normal, (h) gap perturbation Δ*d* = 40 nm and (i) perturbation Δ*d* = 40 nm, Δ*w* = −20 nm. The black and red curves represent the results for the input TE_0_ and TM_0_ mode, respectively.

As a proof of concept, we also fabricated a device to demonstrate such mode controlled TES. The device was fabricated on a 350-nm silicon on insulator with same preset parameters as in the simulations. The scanning electron microscope (SEM) images are shown in Figure 4(a)–(c), corresponding to the three configurations: normal (Figure 4(a)), gap deviated (Figure 4(b), Δ*d* = 40 nm), both gap and width deviated (Figure 4(c), Δ*d* = 40 nm and Δ*w* = −20 nm).

In our experiment, two edges of the SSH waveguide lattice act as the two channels for the waveguide TE_0_ and TM_0_ mode separately. The input TE_0_ mode remains in the excited edge channel, whereas the TM_0_ mode is directionally coupled to the other edge channel. The tested wavelengths range from 1530 nm to 1566 nm with the interval of 4 nm, covering almost the whole C-band. Figure 4(d) shows the experimentally tested efficiency curves in both edge channels of the device. The efficiencies for the desired mode is dominant in the whole tested wavelength range in each channel, with both approximately 10-dB mode-extinction ratios (the power ratio between the TE_0_ and TM_0_ mode) near the wavelength of 1550 nm, indicating the successful mode-controlled TES.

For the structure perturbative configuration with Δ*d* = 40 nm, the experimental results of the tested efficiencies are similar to the results of the normal one, as shown in Figure 4(e). However, for the perturbative configuration with Δ*d* = 40 nm and Δ*w* = −20 nm, the tested efficiencies for both edge channels are relatively lower than those in the normal configuration. Nevertheless, the experimentally tested results still exhibit mode-controlled TES with noticeable mode extinction ratios in the entire wavelength range (Figure 4(f)). The low efficiencies observed in the tested TM_0_ edge channel (Figure 4(d)–(f)) may be attributed to inadequate coupling between the waveguides, which is caused by unavoidable deviations during the device fabrication process (as shown in Figure S2 in the Supplementary Materials). As a contrast, we also fabricated three conventional double-waveguides structures with the same structure deviations as in Figure 4(a)–(c). The tested efficiency curves are presented in Figure 4(g)–(i), demonstrating the similar results compared to the SSH waveguide lattice (Figure 4(d)–(f)).

## 5 Discussions

In conclusion, this work has successfully demonstrated a reconfigurable TES controlled by the waveguide TE_0_ and TM_0_ modes in one-dimensional SSH waveguide lattice. The efficiency curves obtained from the experimental tests have shown that the device operates effectively across the entire C-band, with an approximate mode extinction ratio of 10 dB near the designated wavelength of 1550 nm. It is important to acknowledge that the choice of the gap distance *d*_2_ falls within the sensitive coupling region, where the coupling coefficient undergoes significant variations, as depicted in Figure 2(a). Consequently, the corresponding coupling length also experiences drastic changes. To enhance robustness, it is imperative to explore better parameter combinations that meet the requirements for both robustness and a large DOC value between the modes. By doing so, the designed device can operate as an effective mode splitter, offering performances superior to those of conventional double-waveguides configurations. The findings presented in this work open up new possibilities for manipulating TES and pave the way for numerous applications in integrated topological photonics. Future research could focus on refining the device parameters to improve robustness and further exploring novel applications that leverage the reconfigurable nature of the TES. Exciting advancements in the field of integrated topological photonics are anticipated as a result of these endeavors.

## Supplementary Material

Supplementary Material Details
