# Supplementary material for: Optical mode-controlled topological edge state in waveguide lattice
Source: Nanophotonics. 2024 Jan 23;13(3):319–25. doi: 10.1515/nanoph-2023-0680 (PMC11501731; doi:10.1515/nanoph-2023-0680)
Supplement: Supplementary file 1 — Supplementary Material Details [file j_nanoph-2023-0680_suppl_001.docx]

Supplementary materials for **‘Optical mode-controlled topological edge state in waveguide lattice’**

*Changyu Zhou^1^, Zhenwei Xie^1,*^, Ting Lei^1^, Yao Zhang^2^, Qinmiao Chen^2^, Xiaocong Yuan^1,3*^*

^1^Nanophotonics Research Center, Institute of Microscale Optoelectronics & State Key Laboratory of Radio Frequency Heterogeneous Integration, Shenzhen University, Shenzhen 518060, China

^2^State Key Laboratory on Tunable Laser Technology, Ministry of Industry and Information Technology Key Lab of Micro-Nano Optoelectronic Information System, Harbin Institute of Technology (Shenzhen), Shenzhen, China.

^3^Research Institute of Intelligent Sensing, Research Center for Humanoid Sensing, Zhejiang Lab, Hangzhou 311100, China

^*^Correspondence: ayst31415926@szu.edu.cn; xcyuan@szu.edu.cn

**S1. Analysis for the SSH waveguide lattice**

In the waveguide array based SSH lattice, the optical field located in the waveguide array can be described by the tight-binding coupled-mode equations [1]

$\begin{matrix} -i\frac{d}{dy}\psi_{A,n}=\beta_{0}\psi_{A,n}+\kappa_{1}\psi_{B,n}+\kappa_{2}\psi_{B,n-1}, \\ -i\frac{d}{dy}\psi_{B,n}=\beta_{0}\psi_{B,n}+\kappa_{1}\psi_{A,n}+\kappa_{2}\psi_{A,n+1}, \end{matrix}$ (S1)

where *ψ_A_*,*_n_* (*ψ_B_*,*_n_*) is corresponding localized optical field at the *n*-th A-site (B-site) waveguide. *β*_0_ is the propagation constant of the field in a single waveguide. κ_1_ and κ_2_ are the coupling coefficients between the neighbor waveguides. Optical field is propagated along the *y* direction.

The corresponding Hamiltonian for the SSH lattice is

$H\left(k\right)=\left(\begin{matrix} & \kappa_{1}+\kappa_{2}e^{-ik} \\ \kappa_{1}+\kappa_{2}e^{ik} & \end{matrix}\right),$ (S2)

where *k* is the Bloch quasi momentum. The calculated two bands for the Hamiltonian Eq. (S2) are separated by the magnitude of 2δ = 2|κ_1_−κ_2_|. The winding number of this system can be calculated by $W=\frac{i}{2\pi}\int_{BZ} \langle u_{n}\left(k\right)|\partial_{k}|u_{n}\left(k\right)\rangle dk)$, where *u_n_*(*k*) is the Bloch wave functions. It shows the system is topologically trivial (W = 0) for κ_1_>κ_2_, while is topologically nontrivial (W = 1) for κ_1_<κ_2_.

In this work, an even N-sites SSH waveguide-lattice model is employed, the corresponding Hamiltonian for Eq. (S1) (*β*_0_ = 0 for simplicity) can be rewritten as

$H=\left(\begin{matrix} \begin{matrix} 0 & \kappa_{1} \\ \kappa_{1} & 0 \end{matrix} & \begin{matrix} 0 & \\ \kappa_{2} & \end{matrix} & \begin{matrix} & \\ & \end{matrix} \\ \begin{matrix} 0 & \kappa_{2} \\ & \end{matrix} & \ddots& \begin{matrix} & \\ & \end{matrix} \\ \begin{matrix} & \\ & \end{matrix} & \begin{matrix} & \\ & \end{matrix} & \begin{matrix} 0 & \kappa_{1} \\ \kappa_{1} & 0 \end{matrix} \end{matrix}\right)_{N\times N}$ (S3)

The band structure can be calculated by the above Eq. (S3), as shown in Fig. 1(b) in the main text.

One of the key information is the emergent topological edge state (TES) at the nontrivial phase for κ_1_<κ_2_. There are two near-zero modes localized mostly at the two edges of the waveguide array (*n* = 1 and *n* = N), as shown in Fig.1(b-d) in the main text. The effective coupled length (CL) L_C_ between the two TESs can be simply calculated by L_C_ = π/β_d_, where β_d_ =β_1_-β_2_ is the difference of coupling between the two TESs. It is worth noting that the TES can be controlled by tailoring the difference of coupling β_d_.

**S2. Robustness of the SSH waveguide lattice**

The designed CL is L_C_ = 230 μm in this work, by which the TM_0_ mode is directionally coupled to the other edge mostly, whereas the TE_0_ mode still resides in the original edge channel, and thus the TE_0_ mode is barely affected by the structure perturbations. The robustness (stability of the CL) of the coupled TM_0_ mode is dependent on the gap-distance dependent parameter κ_edge_(*d*), which decides the coupled efficiency for the TM_0_ mode. Fig. S1 shows the theoretically calculated efficiency map for the TM_0_ mode coupled to the other edge with the simulated coupled coefficients. The efficiency is sampled at the designed CL L_C_ = 230 μm with varying the gap distance d_1_ and d_2_. It shows the varying parameters d_1_ and d_2_ should be positively correlated to preserve a high coupled efficiency.

**
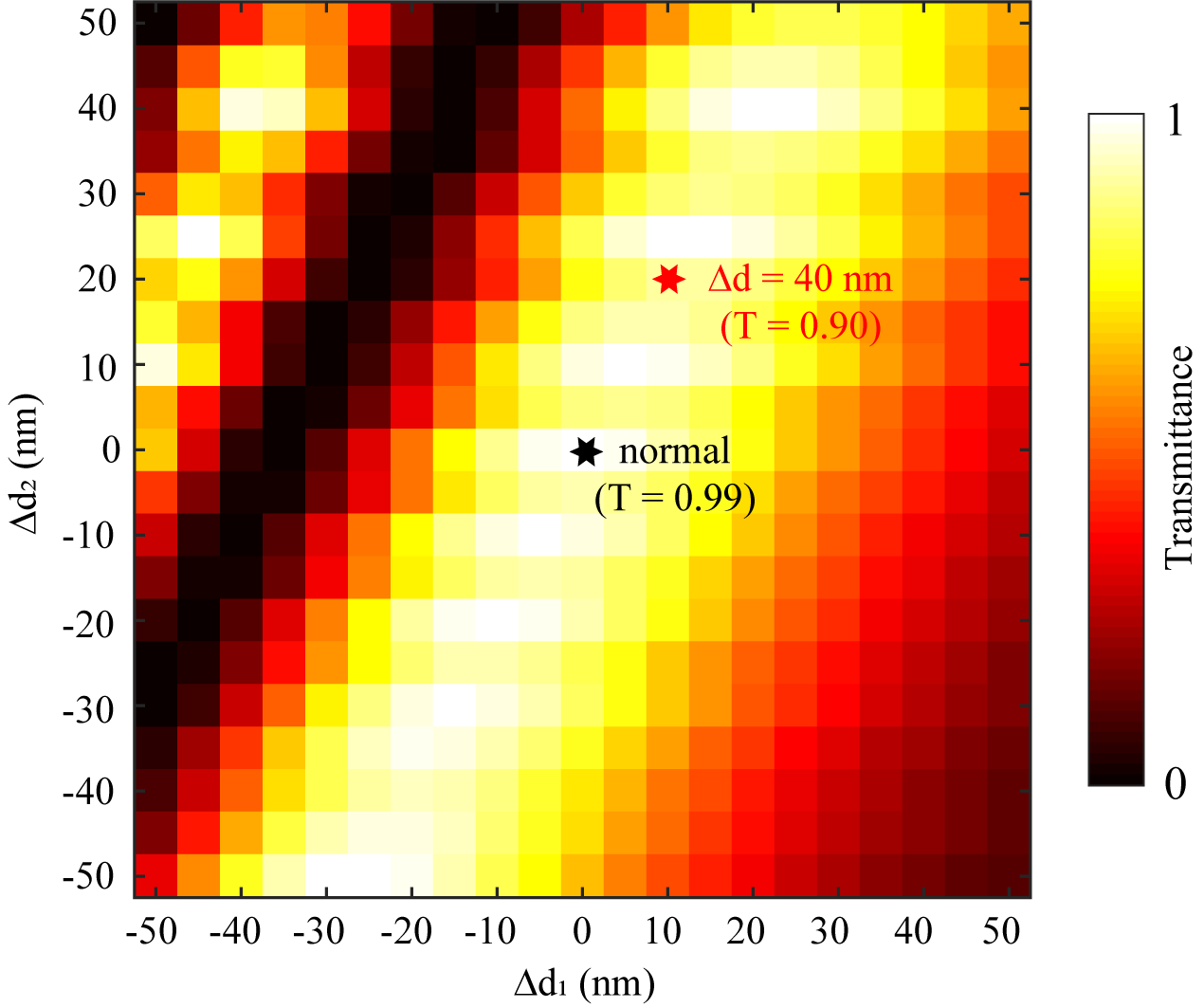
**

**Figure S1.** Theoretically calculated efficiency map for the waveguide TM_0_ mode at the sampled distance of L_C_ = 230 μm. Δd*_i_* is defined as described in the main text, and the black and red stars in the map represent the non-perturbative normal and gap-perturbative configurations used in this work, respectively.

**S3. Device fabrication**


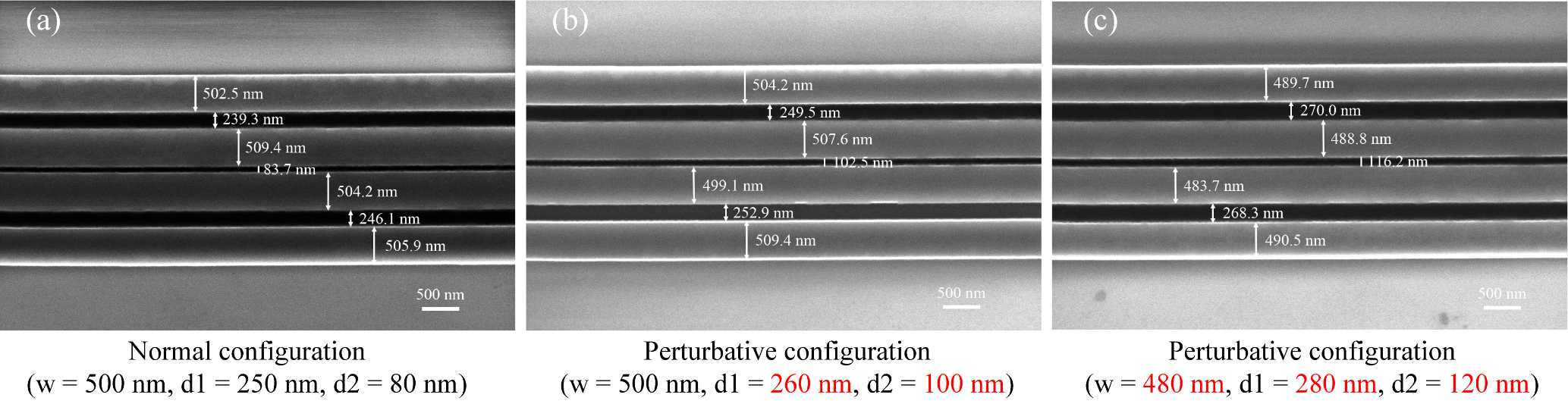


**Figure S2.** The scanning electron microscope (SEM) images of the four-sites SSH waveguide lattice. (a-c) SEM images for (a) normal configuration, (b) perturbative configuration with Δd = 40 nm, (c) perturbative configuration with Δd = 40 nm and Δw = −20 nm.

The devices were fabricated on a silicon on insulator (SOI) wafer, which consists of a 350-nm-thick device layer and a 2-μm-thick buried oxide layer. The fabrication process involved several steps. Firstly, the SOI wafer was spin-coated with a 350-nm-thick layer of ZEP-520A positive resist. It was then baked on a hot plate at 180℃ for 3 minutes. Next, the deigned pattern was exposed by the electron beam photolithography using the Raith EBPG 5150. Subsequently, the exposed sample was developed in the n-Amyl acetate for 1 min and then fixed in the IPA solution for 30 seconds at room temperature. Finally, reactive ion etching was employed to remove the undesired silicon layer, with the SiO_2_ layer of the wafer severs as the etching stop. The fabricated samples have a maximal width (gap) error of approximately 10 nm, as depicted in Fig. S2.

**S4. Experimental test**


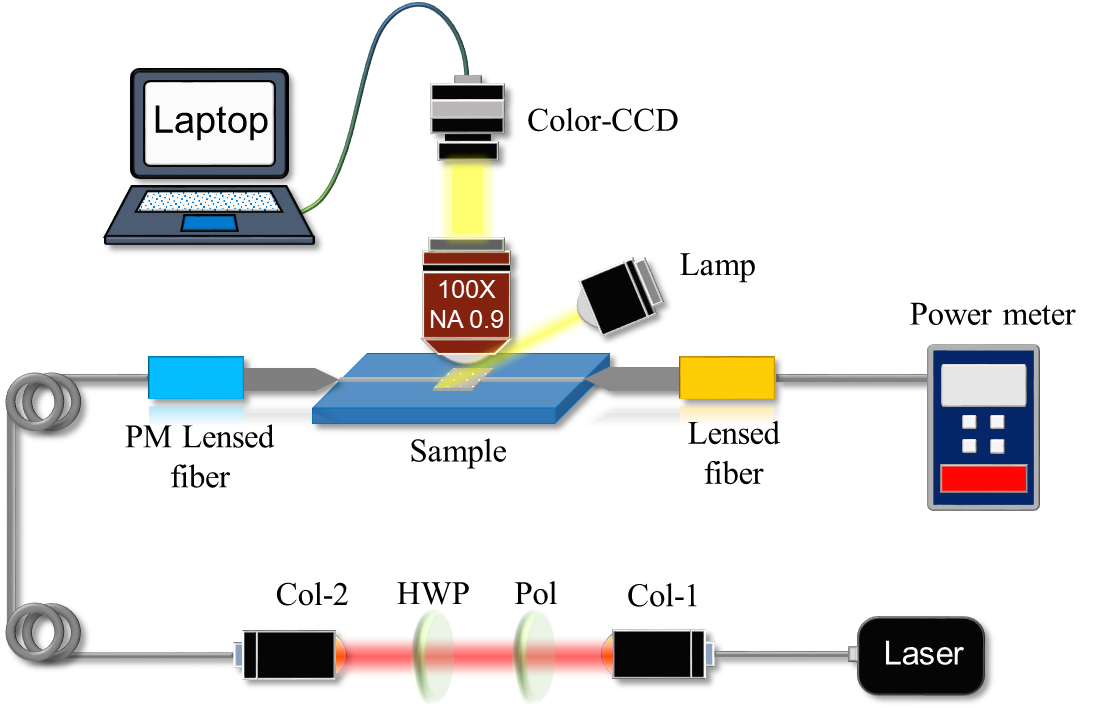


**Figure S3.** Experimental setup. PM: polarization maintained, Col: collimator, HWP: half wave plate, Pol: polarizer.

In the experimental test, the fabricated device underwent test by directly measuring the output intensity using two coupled lensed fibers. To excite the desired input mode (TE_0_ or TM_0_) in the waveguide, the state of polarization was modulated in free space using a combination of a polarizer (Pol) and a half-wave plate (HWP). The modulated light was then coupled to a polarization-maintaining (PM) lensed fiber, ensuring the preservation of polarization states. By simply rotating the HWP, the desired TE_0_/TM_0_ mode could be excited in the waveguide. Subsequently, the output light from the device was coupled to another lensed fiber and recorded using a power meter (Fig. S3). The measured efficiencies were calibrated using a reference straight waveguide to account for any coupling loss between the lensed fiber and the waveguide, as well as the transmission loss within the waveguide itself. The results are presented in Fig. 4 of the main text.

**References**

[1] W. Song; W. Sun; C. Chen*, et al.*, Robust and Broadband Optical Coupling by Topological Waveguide Arrays. *Laser & Photonics Reviews,* *14* (2), 1900193, 2020.
